# Slower Is Higher: Threshold Modulation of Cortical Activity in Voluntary Control of Breathing Initiation

**DOI:** 10.3389/fnins.2018.00663

**Published:** 2018-10-11

**Authors:** Pierre Pouget, Etienne Allard, Tymothée Poitou, Mathieu Raux, Nicolas Wattiez, Thomas Similowski

**Affiliations:** ^1^UMRS 975, INSERM, CNRS 7225, Institute of Brain and Spinal Cord, UPMC - University Pierre and Marie Curie, Paris, France; ^2^UMRS1158, INSERM, Neurophysiologie Respiratoire Expérimentale et Clinique, Sorbonne Universités, UPMC - University Pierre and Marie Curie, Paris, France; ^3^Service de Pneumologie et Réanimation Médicale (Département “R3S”), AP-HP, Groupe Hospitalier Pitié-Salpêtrière Charles Foix, Paris, France

**Keywords:** motor control, countermanding task, breathing, decision making, inhibition (psychology)

## Abstract

Speech or programmed sentences must often be interrupted in order to listen to and interact with interlocutors. Among many processes that produce such complex acts, the brain must precisely adjust breathing to produce adequate phonation. The mechanism of these adjustments is multifactorial and still poorly understood. In order to selectively examine the adjustment in breath control, we recorded respiratory-related premotor cortical potentials from the scalp of human subjects while they performed a single breathing initiation or inhibition task. We found that voluntary breathing is initiated if, and only if, the cortical premotor potential activity reaches a threshold activation level. The stochastic variability in the threshold correlates to the distribution of initiation times of breathing. The data also fitted a computerized interactive race model. Modeling results confirm that this model is also as effective in respiratory modality, as it has been found to be for eye and hand movements. No modifications were required to account for respiratory cycle inhibition processes. In this overly simplified task, we showed a link between voluntary initiation and control of breathing and activity in a fronto-median region of the cerebral cortex. These results shed light on some of the physiological constraints involved in the complex mechanisms of respiration, phonation, and language.

## Introduction

In vertebrate animals, the central nervous system generates a rhythmic command that drives the contraction of respiratory muscles in order to move air in and out of the lungs. The mechanisms of this automatic control have been deeply investigated. This control relies primarily on groups of brainstem neurons in dynamic interaction (Feldman et al., 2013) that generate the respiratory rhythm and adjust it to the metabolic activity of the body. Voluntary breathing commands can also arise from higher brain structures, and the respiratory muscles are represented within the primary motor cortex (Smith, 1938; Gandevia and Rothwell, 1987; Similowski et al., 1996). Patients with locked-in syndrome retain emotional influences on breathing but have no voluntary control of respiratory movements (Heywood et al., 1996). Over the last decade, functional studies in humans have shown that neuronal activity in the premotor cortex and the supplementary motor areas are also involved when subjects are exposed to inspiratory resistance and breathe against it without being instructed to do so (Raux et al., 2007, 2013). Cortico-subcortical cooperation in generating the neural drive to breathe has been demonstrated in patients with deficient respiratory automatism (Tremoureux et al., 2014a), in normal subjects during hypocapnia-related inhibition of the respiratory automatism (Dubois et al., 2016), in patients with inspiratory muscle weakness (Georges et al., 2016), and in patients with abnormally high inspiratory resistances (Launois et al., 2015). In all cases their electroencephalographic activity suggests involvement of the premotor cortex. In this study we pushed the argument further by testing the hypothesis of a causal involvement of the cerebral cortex in the voluntary initiation and inhibition of a single breath command, and tested it by recording respiratory-related premotor cortical potentials in the scalp of awake subjects during such maneuvres.

Although, research on voluntary respiratory control is most often based on neurophysiological studies, computational modeling also has a role to play. Among the multitude of existing computational models, the race model offers an interesting method to investigate breathing modality in the context of an inhibitory task. This task consists of two types of trials: Go trials, where subjects were instructed to perform an action as quickly as possible, and Stop trials, where subjects had to inhibit this action. This paradigm is useful when studying the ability of a subject to inhibit an action, and allows to the time needed to stop an action to be assessed (Stop Signal Reaction Time or SSRT), which is not observable directly. Logan et al. (1984) developed a race model to estimate the SSRT in an oculomotor countermanding task. Beyond the access to the time needed to cancel an action, this model provides a way to explore functional mechanisms responsible for inhibition performance. This model involves two independent units, a go and a stop process, performing a race from a baseline until one of these processes crossed an arbitrary threshold; the winner of this race is the first process that crosses the threshold. More recently, Boucher et al. (2007a) added interactions between go and stop units to account for electrophysiological recordings of single units in macaques. Race models have been tested in eye and hand modality (Boucher et al., 2007b) but never, to our knowledge, on respiratory modality. In this study, we tested the modified Boucher et al.’s race model to assess whether it would be qualitatively adequate to account for respiratory data in the particular context of a countermanding task.

Our study focused on the magnitude and timing of the fronto-median cortical premotor potential activity, examining whether its stochastic variability could account for breathing initiation time and inhibition. This study is an important contribution to an improved understanding of how humans initiate vocalizations and the disentanglement of the distinct and shared processes within the complex mechanisms of respiration, phonation, and language.

## Materials and Methods

### Subjects and Session Design

This study was part of a wider respiratory-related cortical activity research program that has been approved by the local ethical committee. The subjects gave informed consent to participate. Data were collected from six human subjects (five males, mean age 31 years ± 8). Each subject participated in two sessions with 256 NoStop trials and two sessions with 128 NoStop and 128 Stop trials. Each session lasted for approximately 60 min. The trial order and the session order were both randomized across subjects. All subjects reported having normal or corrected-to-normal vision.

### Ventilatory Movement Recording

The subjects’ ventilatory movements were measured using custom-built magnetometers (Mead et al., 1967). Two magnets were positioned, one ventrally and one dorsally, at the level of the umbilicus using an elastic belt. This allowed us to measure abdominal expansion, a direct indicator of diaphragmatic contraction insofar as the diaphragm is the only muscle whose contraction increases abdominal circumference. A PC with a NI-DAQ analog acquisition card (National Instruments Corp., Austin, TX, United States) running the *Xenomai* operating system for parallel real-time acquisition (sampling frequency 1,000 Hz) recorded ventilatory movements and the various stimuli presented on-screen during the sessions.

### Initiation Breathing Task – NoStop Trials

**FIGURE 1:**
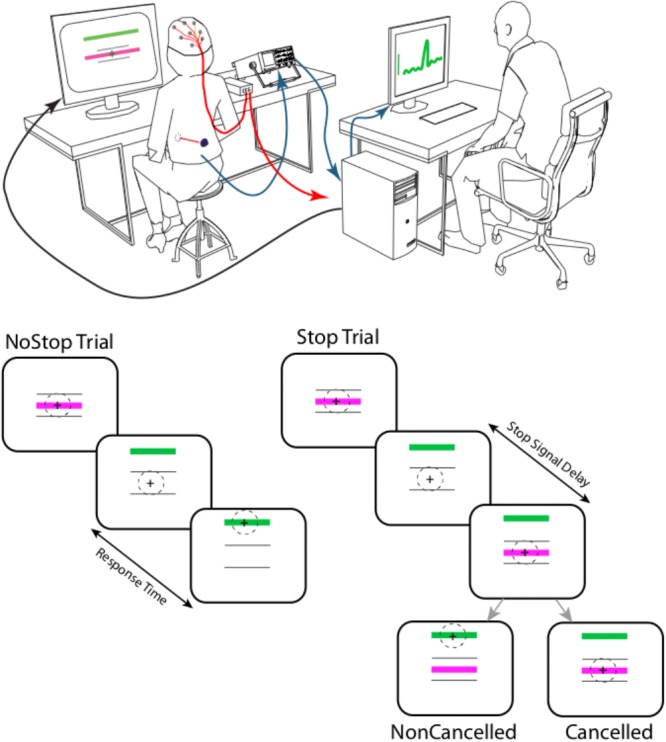
Experimental setup: the respiratory movements of the subjects were assessed using a magnetometer that measured the distance between two magnets. These were placed on the abdomen and on the back at the height of the umbilicus, and maintained in position with an elastic belt. This allowed us to measure abdominal expansion, an indirect indicator of diaphragmatic contraction. During respiratory tasks, electroencephalographic potential (ERPs) signal were recorded. Each trial started with the presentation of two purple horizontal bars (10° length of visual angle) centered on a black background. The two bars were vertically separated by 10° of visual angle. After a random delay ranging from 500 to 1,000 ms, a green bar (Go signal) appeared 6° above the top fixation bar, instructing the subject to initiate an inspiration. Abdominal movements were displayed on the subject’s screen as a cross that moved up and down in relation to abdominal expansion and contraction to provide feedback to the subject of the amplitude of the evoked respiratory response. On 50% of trials (at random), after a delay (Stop Signal Delay- SSD) ranging from 48 to 640 ms, a green bar was presented in the center of the screen, instructing the subject to stop the inspiration. A failure to inhibit inspiration was classed as a non-canceled trial, while a successfully inhibited inspiration was classed as a canceled trial.

Subjects sat 57 cm away from a TV display monitor (Dell 21”). Each trial started with the presentation of two purple horizontal bars centered on a black background. The two bars were vertically separated by 10° of visual angle (**Figure 1**). After a random delay ranging from 500 to 1,000 ms, a green bar (Go signal) appeared 6° above the top fixation bar, instructing the subject to initiate inspiration. To provide feedback of the amplitude of the corresponding evoked respiratory response, the subject’s abdominal movements were displayed on the screen as a cross that moved up and down with abdominal expansion (see below). A calibration procedure was performed at the beginning of each block to adjust gain and offset so the respiration amplitude modulation remained within the range of the breathing initiation bars.

### Countermanding Breathing Task – Stop Trials

As in the initiation-breathing task, each trial started with the presentation of two purple horizontal bars centered on a black background (**Figure 1**). The two bars were vertically separated by 10° of visual angle. After a random delay ranging from 500 to 1,000 ms, a green bar (Go signal) appeared 6° above the top fixation bar, instructing the subject to initiate an inspiration. Abdominal movements were displayed on the subject’s screen as a cross that moved up and down according to abdominal expansion and contraction to provide feedback to the subject of the amplitude of the evoked respiratory response. A calibration procedure was performed at the beginning of each block to adjust gain and offset so the respiration amplitude modulation remained within the range of the breathing initiation bars. In a second type of trial – the task started with the presentation of two purple horizontal bars centered on a black background. On 50% of trials (at random), after a delay (Stop Signal Delay- SSD) ranging from 48 to 640 ms, a green bar was presented in the center of the screen, instructing the subject to stop his/her inspiration. A failure to inhibit inspiration was classed as a non-canceled trial, while a successfully inhibited inspiration was classed as a canceled trial.

### Reaction Time Measurement

The ventilatory movement signal was processed offline using Matlab (MATLAB Release 2012b, The MathWorks, Inc., Natick, MA, United States). The onset of breathing movement was determined using a derivative of the abdominal expansion signal based on a threshold limit compared with the resting breathing signal. The reaction time was the difference between a Go signal presentation and the onset of inspiration.

### EEG Signal Recording

During respiratory tasks, electroencephalographic (EEG) signal was recorded using nine active electrodes positioned according to the international 10–20 system, recorded with a V-Amp system, (Brain Product, Munich, Germany). The reference was calculated from the electrodes A1 and A2. The impedance of each electrode was estimated between 5 and 10 kΩ and was always lower less than 25 kΩ. Abdominal ventilatory movements and the EEG signal were later synchronized using markers.

### EEG Signal Processing

Ensemble averaging was first performed to improve the signal-to-noise ratio and reveal the potentials, in a manner typical to the study of evoked potentials. The continuously recorded electroencephalographic signal was split into three epochs, each of one second, extending from 0.5 s before to 2.5 s after the Go signal presentation (green bar). A thresholding method was used to detect artifact and periods exhibiting activity ± 3 standard deviation of the mean were discarded. The rejection rate was approximately 30% in the various sessions. Trials were sorted into five (NoStop sessions) or three (NoStop trials of Stop sessions) groups according to reaction time, and the EEG signal was averaged point by point.

### Race Model

The interactive race model is composed of a go unit and a stop unit. Their activity is governed by two stochastic differential equations (Usher and McClelland, 2001) with a null leakage factor:

(1) $${\mathrm{da}}_{\mathrm{go}}(t)=\mu_{\mathrm{go}}-{ß}_{\mathrm{stop}}.a_{\mathrm{stop}}(t)+\xi_{\mathrm{go}}$$

(2) $${\mathrm{da}}_{\mathrm{stop}}(t)=\mu_{\mathrm{stop}}-{ß}_{\mathrm{go}}.a_{\mathrm{go}}(t)+\xi_{\mathrm{stop}}$$

Each unit is defined by three parameters: the mean growth rate (μ); the inhibition parameter on the other unit (ß); and a Gaussian noise term (*ξ*) with a mean of zero variance of σ^2^_go_ or σ^2^_stop_, where *a* represents the activity of the unit. The race finished when a unit crossed the threshold, fixed at 1,000 (arbitrary units), within a limit of 800 ms. If unit activity is negative during the race, the activity was reset to zero at this point (non-physiologic value). An additional parameter, D, was added to the model to take into account the stimulus encoding that occurred in the go and stop units (respectively D_go_ and D_stop)_. D_go_ was determined and set constant for each subject using values from the EEG recordings. D_go_ was calculated when the activity signal equaled the baseline mean plus five standard deviations in the EEG signal. D_stop_, μ, ß, and σ are the unconstrained parameters of the model.

We tested these interactive race models to find the parameters that best fitted the data from six subjects who performed two sessions of a breathing countermanding task. Two sessions were removed from the analysis because of bimodality observed in reaction times.

We fitted inhibition function, reaction time distributions of correct NoStop trials and failed stop-signal trials. For each fit, we computed a chi-square test between data from the model and the subjects. Inhibition function chi-squares were calculated by summing chi-squares computed at each stop-signal-delay (SSD) between error rates from model and subject data. The chi-squares of the reaction times of correct and failed NoStop stop-signal trials were calculated as follows: each distribution was sorted into five quintiles; a “local” chi-square was computed at each quintile between the proportion of trials from model regarding subjects’ data, then the five “local” chi-squares were added. A general chi-square was then calculated by adding the local chi-squares together. To find the best parameters for each subject, we minimized the general chi-square using a minimization function (*patternsearch* from the global optimization toolbox of Matlab). *Patternsearch* looks for a minimum based on an adaptive *mesh* that is aligned with the coordinate directions. Because minimization functions are sensitive to start point, we ran *patternsearch* from 50 randomly chosen starting points. Finally, to avoid a local minimum, we again started *patternsearch* from 200 new start points. These starting points were determined by the best parameters from first run and were defined as follows: best parameters ± 0.01 units and best parameters ± 0.02 units. All these procedures were performed on a supercomputer cluster (NEC, 40 nodes, 28 cpu Intel Xeon E5-2680 V4 2.4 GHz/node, 128 Go RAM/node).

## Results

**FIGURE 2:**
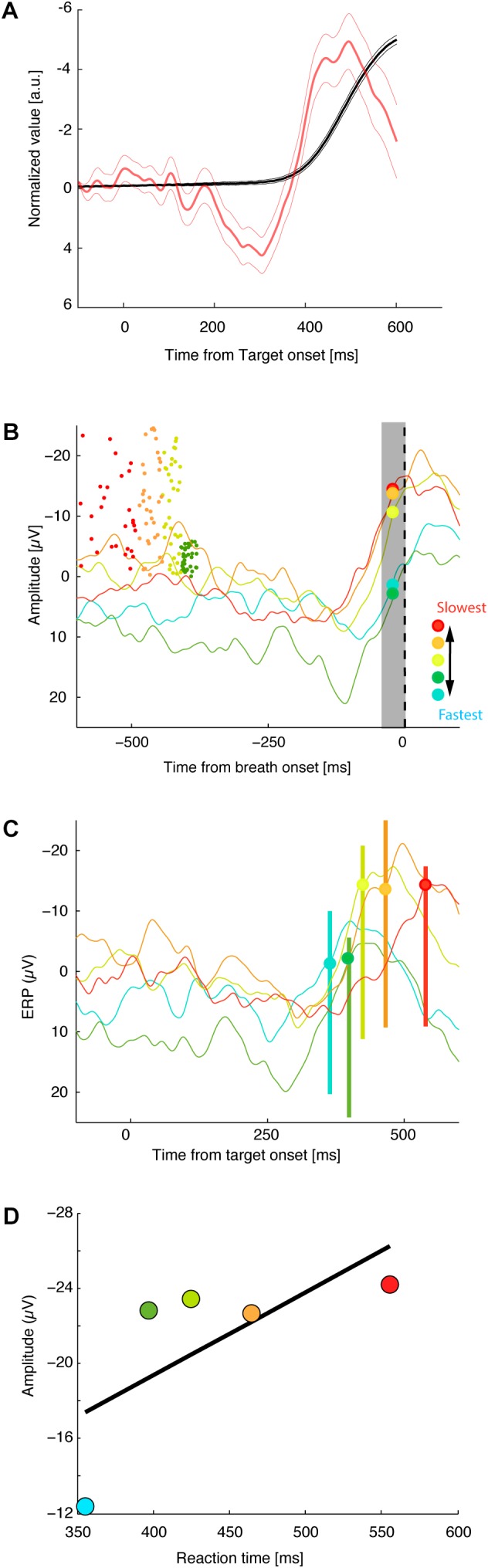
Threshold of premotor activity response as a function of breath initiation times. **(A)** Normalized average of the diaphragmatic signal (black lines) and normalized activity at Fz aligned on target onset (red lines). Thick and thin vertical lines represent, respectively, the mean of response times ± standard error of mean. **(B)** Average activity at Fz aligned on breath onset. Dot indicates the time of target presentation of each correct trial (the Y values of each dot represents, its trial position during the session). Trials have been sorted by mean reaction time into five groups. **(C)** Activity at FZ aligned on target onset (same convention as in **B**). **(D)** Average activation level 10–20 ms before breath initiation is plotted against mean reaction time for the five reaction time groups for this recording session.

**Figure 2** shows the responses from a representative session of 256 NoStop trials. The activity recorded at *Fz* began to increase ∼100 ms before breathing initiation, peaking shortly before breathing initiation.

**FIGURE 3:**
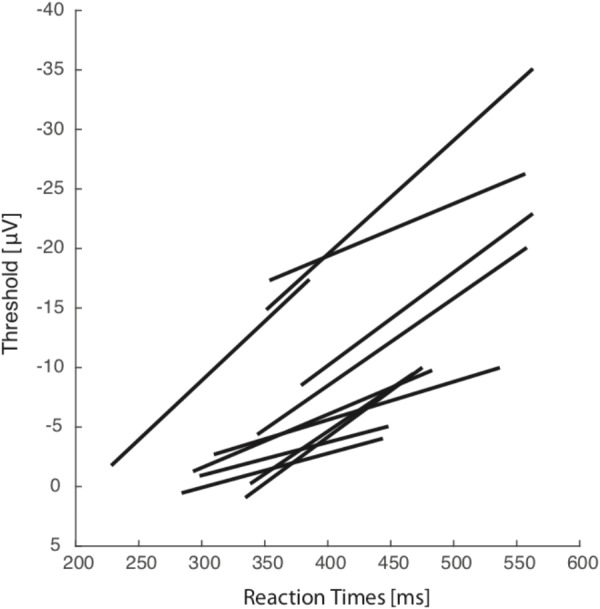
Across all sessions and all subjects. Average activation level 10–20 ms before breath initiation is plotted against mean reaction time for five reaction time groups representative of each particular session. Each line represents the linear regression plot between reaction time.

Specific measures of movement-related neural activity were required to evaluate the prediction that the trigger threshold of breathing preparation varied with reaction time. We tested this prediction by measuring the level of neural activation as a function of the time at which the presumed threshold triggering the movement was crossed. On the basis of electrophysiological studies of cortical control in arm, leg, and eye movements, we estimate that measurements of neural activity 10–20 ms before breathing initiation are an accurate index of the level of trigger threshold activation. We defined the threshold activation as the average level of the activation function in the period between 20 and 10 ms before breathing initiation. We compared the activation threshold across groups of NoStop-trials with different reaction times. **Figure 2** presents the activity at *Fz* for sorted response times aligned on breath onset (top panel) and target presentation (middle panel). The activation threshold increases for longer reaction times (bottom panel). We divided the distribution of trials into five groups according to their reaction times (Neshige et al., 1988). A linear regression analysis indicated a significant relation between the activation threshold and reaction time. As shown in **Figure 3**, significant changes in activation threshold with reaction time were observed for 11 of the 12 sessions (*R*^2^ = 0.78 ± 0.21, mean and standard error, all *p*-values were lower than 0.001). The p-value was computed by transforming the correlation to create a t-statistic having 3° of freedom. The confidence bounds were based on an asymptotic normal distribution of 0.5^∗^*log*((1 + r)/(1 – r)), with an approximate variance equal to 1/(N – 3).

In one session, the impedance value was too high and signal quality was insufficient to be included in the analysis. These results are consistent with variable threshold activity in adjustment of reaction time in breathing.

**FIGURE 4:**
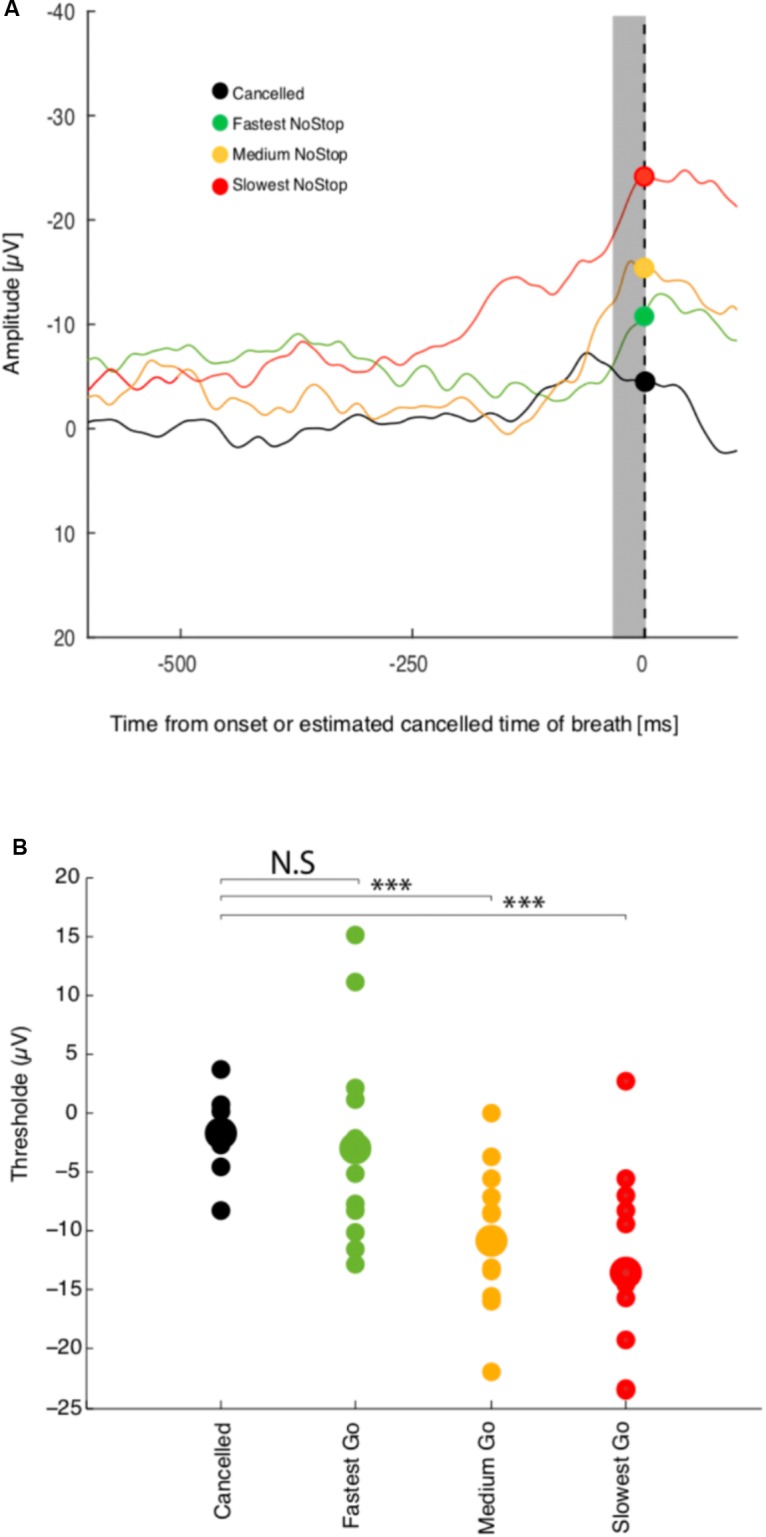
Threshold premotor activity response and breathing inhibition or breathing initiation times. **(A)** Activity at Fz aligned on breath onset. NoStop trials have been sorted by mean reaction time into three groups. Activity recorded during successful Canceled trials are presented by the black curve **(B)** Average activation level 10–20 ms before breath initiation or estimated response time are plotted against mean reaction time for the three reaction time groups for this recording session. ^∗∗∗^Represents significant *p* value < 0.001.

To determine not only if activation threshold co-varies with reaction time, but if this activity is sufficient to predict whether breathing is going to be initiated or not, we examined the modulation of activity recorded at *Fz* while the subject was performing a countermanding breathing task (**Figure 1**). **Figure 4A** shows the response of a representative session of 256 non-canceled trials and 256 canceled trials. A linear regression analysis indicates a relation between the threshold activation and reaction time (*R*^2^ = 0.48, *p* < 0.001) for this session.

As in the NoStop task, *Fz* activity in the non-canceled trials recorded began to increase approximately 100 ms before breathing initiation, peaking shortly before it, which we characterize as a failure of inhibition. We compared the activation threshold across groups of non-canceled trials with the activity during canceled trials. The activity in canceled trials remained low and never reached the threshold activity of trials with shorter or longer reaction times as shown in **Figure 4** (top panel). The threshold activation for the group of canceled trials was essentially unchanged compared to baseline activity. Changes in threshold activation with reaction time were observed between subjects: see **Figure 4** (bottom panel). These results are consistent with variable threshold activity and causal linkage between premotor threshold activity and breathing initiation.

**FIGURE 5:**
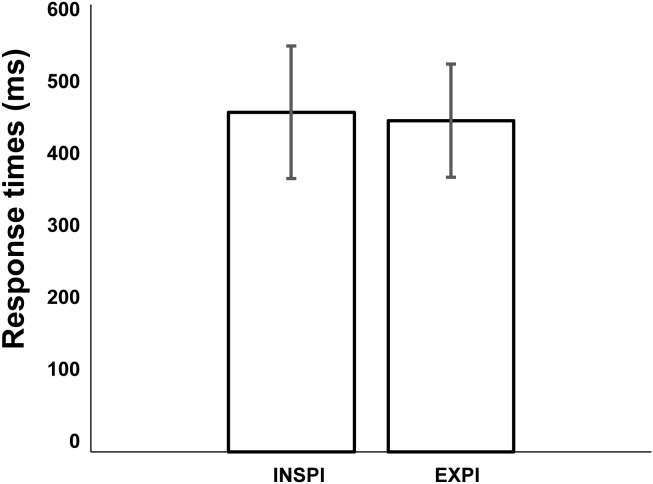
Comparison of mean response times initiated in the half cycle including the initiation of inspiration movement or half cycle including the initiation of expiratory movement (Mann–Whitney *U*-test: *p* = 0.79). Error bars represent the standard deviation of the mean (across 6 subjects and 11 sessions).

To compare response time according to the phase of the spontaneous cycle we divided the response trials into two groups. In the first group, defined as the Inspiratory group, the response times were made in the half cycle that included the initiation of inspiration movement. In the second group, defined as the Expiratory group, the response times were made in the half cycle that included the initiation of expiratory movement. Across sessions and subjects (**Figure 5**), the average mean response times initiated in the half cycle including the initiation of inspiration movement was not significantly different from the response times initiated in the half cycle that included the initiation of expiratory movement (Mann–Whitney *U*-test: *p* = 0.79).

The outputs of data modeling are the best parameters μ (mean growth rate), Ò (noise in simulated signals), ß (weight of inhibition of the other unit), and D_stop_ (time to encode stop signal) corresponding to the smallest find by minimization procedure.

Results from behavioral data modeling by interactive race model are summarized in **Table 1** for each subject and session. SSRT_s_ represents the Stop Signal Reaction Time (ms) calculated by an integration method (Hanes and Schall, 1995) based on simulated data.

**Table 1:** Outputs of data modeling for each behavioral session.

Subject/Session	1a	1b	2a	2b	3a	3b	4a	5a	5b	6a
μ_go_	0.91	1.64	2.71	2.34	1.96	1.4	1.97	2.37	2.31	2.34
Ò_go_	36.82	46.05	16.51	10.99	11.63	15.53	6.29	23.79	11.96	15.34
μ_stop_	45.95	17.5	81.72	8.58	66.35	79.69	77.29	81.76	64.68	83.13
Ò_stop_	50.81	32.22	99.28	81.81	83.16	85.24	54.01	74.41	22.8	97.19
ß_go_	6.37	9.07	0.51	0.33	0.36	0.35	0.05	0.37	0.13	0.41
ß_stop_	3.25	3.49	6.39	8.5	5.56	4.63	5.61	5.89	6.48	3.18
D_stop_	93	88	164	152	30	55	161	116	145	126
χ^2^	0.06	0.11	0.07	0.27	0.06	0.26	0.12	0.08	0.06	0.25
SSRT_s_	58	206	333	329	196	132	179	236	234	236

**FIGURE 6:**
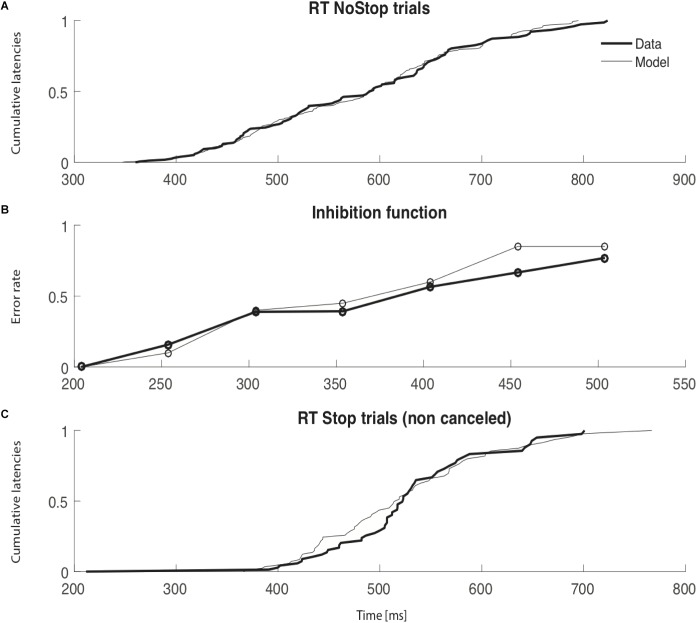
Thin lines represent simulated data and thick lines observed data. For each panel, the *x* axis is time (ms). **(A)** Cumulative latencies of NoStop trials. **(B)** Inhibition function. The *y* axis represents the error rate in response to Stop trials and the *x* axis the times of the stop signal delay (SSD). **(C)** Cumulative latencies of non-canceled Stop trials.

An example of simulated data compared to observed data is shown in **Figure 6** to illustrate the model fitting. This figure represents real data from session 3a and the best parameters from the interactive race model. Panel a) shows the cumulative latencies of NoStop trials; here we can see that these parameters of the model qualitatively fit the real distribution of reaction times. Panel b) shows the inhibition function; the simulated data closely resemble the observed experimental data though when stop signal delay was 450 ms, there was some divergence. Panel c) shows the cumulative distribution of non-canceled Stop trials; again, modeled data closely resemble experimental data, apart from between 400 and 500 ms. In all but one session (session 1a) the initiation of an inhibition curve was observed for SSDs shorter than 200ms (see **Supplementary Figure 1**, for all sessions). In one session (session 1a) the estimation of SSRTs was problematic and therefore require cautious interpretation. Similar outputs and models’ mimicries exist in the context of countermanding task (modulation of e.g., μgo or DSTOP etc…). Therefor possible combinations of parameters might produce similar estimates of behavioral parameters (Pouget et al., 2011). Simultaneous physiological and behavioral measurement would be required to disentangle such possible discrepancies. In our present context, our models were unsufficently constraint to be able to conclude. Our modest goal was simply to expose the fact that a already proposed simple model of eye movement control holds with a unique control of breathing initiation.

## Discussion

Our study focused on specific premotor activity potentials in the fronto-medial cortex that increase in relation to voluntary breathing (Macefield and Gandevia, 1991), loaded breathing (Raux et al., 2007), or speech breathing (Tremoureux et al., 2014b). We first tested whether variability in a single breathing initiation time might be accounted for by modulation of this activity. The results show that voluntary breathing is initiated if, and only if, the cortical premotor potential activity reached a threshold activation level. In the context of countermanding a breathing task, our results show that voluntary breathing is initiated if, and only if, the cortical premotor potential activity reaches a specific threshold activation level. The stochastic variability of this threshold correlates with the distribution of breathing initiation times. Similar premotor potentials have been recorded during voluntary limb, leg and eye movements, with some cerebral potentials being distinguishable in the latter. The major component is a slow negativity, termed the Bereitschaftspotential (readiness potential), which develops before the movement (Deecke et al., 1969; Papakostopoulos et al., 1975; Kristeva and Kornhuber, 1980). Based on subdural recordings from the exposed cerebral cortex (Lee et al., 1986; Neshige et al., 1988), and on topographical analysis of scalp recordings (Barrett et al., 1986; Tarkka and Hallett, 1990), this premotor potential is considered to originate in the supplementary motor area and primary motor cortex. This evoked potential does not accompany pathological limb movements generated subcortically (Obeso et al., 1981). Additionally, this potential is present during an array of cortically controlled respiratory movements (see above), but does not accompany involuntary respiratory activity (Macefield and Gandevia, 1991) or abnormal respiratory activity such as hiccups (Raux et al., 2007). To our knowledge this is the first study that demonstrates that voluntary breathing initiation times might be accounted for by the modulation of cortical premotor potential activity.

Regarding breathing control, several mechanisms are required for adequate speech production. Firstly, as speech is produced during expiration, the control of the duration of vocalized sentences implies inhibition on the automatic breathing pattern generators (for automatic inspiration not to provoke unwanted speech interruptions). In many animals, breath holding during submersion involves strong reflex inhibition of respiratory activity, for example in reaction to snout submersion in the dog (review in Butler, 1982); see also (Lin, 1982). However, natural-diving mammals can perform voluntary apneas spontaneously or in response to training (Ridgway et al., 1969). In humans, a cortical network capable of substantiating such speech-related inhibitory inputs has been described during voluntary apneas (McKay et al., 2008). Secondly, producing sentences of variable length at a variable loudness implies the ability to prepare these sentences through tailored pre-phonatory breaths. We have previously shown that the corresponding respiratory EEG activities were similar to those involved in the production of voluntary respiratory maneuvres such as sniffing (Tremoureux et al., 2014b), suggesting that some aspects of the speech-related breathing control might derive from a previously selected ability to cortically prepare the volume and timing of particular breaths. In certain species, the ability to prepare inspirations according to locomotor and environmental context appears to be crucial. For example, marine mammals must coordinate inspirations with surfacing, sometimes with important timing constraints, such as during sustained rapid swimming in dolphins. Their ability to voluntarily control breathing for non-respiratory purposes has long been described (Ridgway et al., 1969), e.g., during bubble ring play (McCowan et al., 2000). Thirdly, speech must be fluently adapted to social interactions. Participation in conversation implies the possibility to prepare pre-phonatory breaths, to cue them from various signals, to adapt them to unplanned changes in speech programming, or to completely inhibit them to comply with the necessities of the inter-human exchange. This conversational ability requires excitatory-inhibitory interplays very similar to those described for locomotor and oculomotor movements. Our data suggest that this is indeed the case. Furthermore, the interactive race model has been tested on countermanding eye and hand tasks in previous studies (Papakostopoulos et al., 1975), and our results suggest that this model is also qualitatively effective in breath modality without any further modification.

While analyzing possible interactions between the voluntary and reflexive commands of expiration we did not find any statistical differences between reaction times at different points in the cycle of respiration: reaction times during expiration are not significantly longer than in inspiration. Because many factors could lead to an absence of significant variations our results only indicate that any possible interaction between the voluntary and reflexive command, if it exists, is weak in the context of our countermanding paradigm. In support of these findings, it has been shown that the spinal inspiratory motoneurons are hyperpolarized during expiration compared with inspiration (see for example Berger, 1979). Furthermore, it has been also been shown that the automatic inspiratory drive is sufficient to facilitate the response of the diaphragm to the cortical inputs generated by transcranial magnetic stimulation (Straus et al., 2004; Mehiri et al., 2006). It could therefore have been hypothesized that, in our present experiment, RTs would have been shorter for voluntary inspirations initiated within the inspiratory phase of the automatic breathing cycle than within its expiratory phase as a consequence of bulbo-spinal facilitation. The fact that this was not the case is coherent with the notion that the expiratory disfacilitation of respiratory moto-neurons can be overcome by corticospinal inputs in animals (see for example Planche, 1972) as in humans (Similowski et al., 1996). It also indicates that the excitatory corticospinal drive to inspiratory muscles not only bypasses the brainstem central pattern generators (Corfield et al., 1998) but can also be powerful enough to be beyond modulation by their output (under resting breathing conditions). The high values of μ_stop_ and ß_stop_ observed in most of the model sessions underline the necessity for the inhibition process to be very fast to be effective. These observations suggest that the automatic respiration cycle generated by the brainstem can be overridden by cortical outputs with the same efficiency at any moment of the respiratory cycle and that these cortical outputs can take full precedence over their subcortical counterparts.

## Ethics Statement

The research was carried out in accordance with the principles outlined in the Declaration of Helsinki. The subjects gave their written informed consent and the study received the ethical and legal approval of the appropriate external body (Comit de Protection des Personnes Paris Ile de France VI).

## Author Contributions

EA, TP, and PP designed the experiments. EA and TP performed the experiments. EA, PP, and NW analyzed the data. EA, MR, NW, TS, and PP wrote the manuscript. NW computed model part. All authors reviewed the manuscript.

## Conflict of Interest Statement

The authors declare that the research was conducted in the absence of any commercial or financial relationships that could be construed as a potential conflict of interest.

## Supplementary Material

FIGURE S1 Best interactive model predictions across all subjects. The conventions are the same as those of **Figure 5**. Thin lines represent simulated data, thick lines observed data. For each panel, the *x* axis is time (ms). **(a)** Cumulative latencies of NoStop trials. **(b)** Inhibition function. The *y* axis represents the error rate in response to Stop trials and the *x* axis the times of the stop signal delay (SSD). **(c)** Cumulative latencies of non-canceled Stop trials.
