# Supplementary material for: Slower Is Higher: Threshold Modulation of Cortical Activity in Voluntary Control of Breathing Initiation
Source: Front Neurosci. 2018 Oct 11;12:663. doi: 10.3389/fnins.2018.00663 (PMC6193114; doi:10.3389/fnins.2018.00663)
Supplement: FIGURE S1 — Best interactive model predictions across all subjects. The conventions are the same as those of Figure 5. Thin lines represent simulated data, thick lines observed data. For each panel, the x axis is time (ms). (a) Cumulative latencies of NoStop trials. (b) Inhibition function. The y axis represents the error rate in response to Stop trials and the x axis the times of the stop signal delay (SSD). (c) Cumulative latencies of non-canceled Stop trials. [file Data_Sheet_1.PDF]

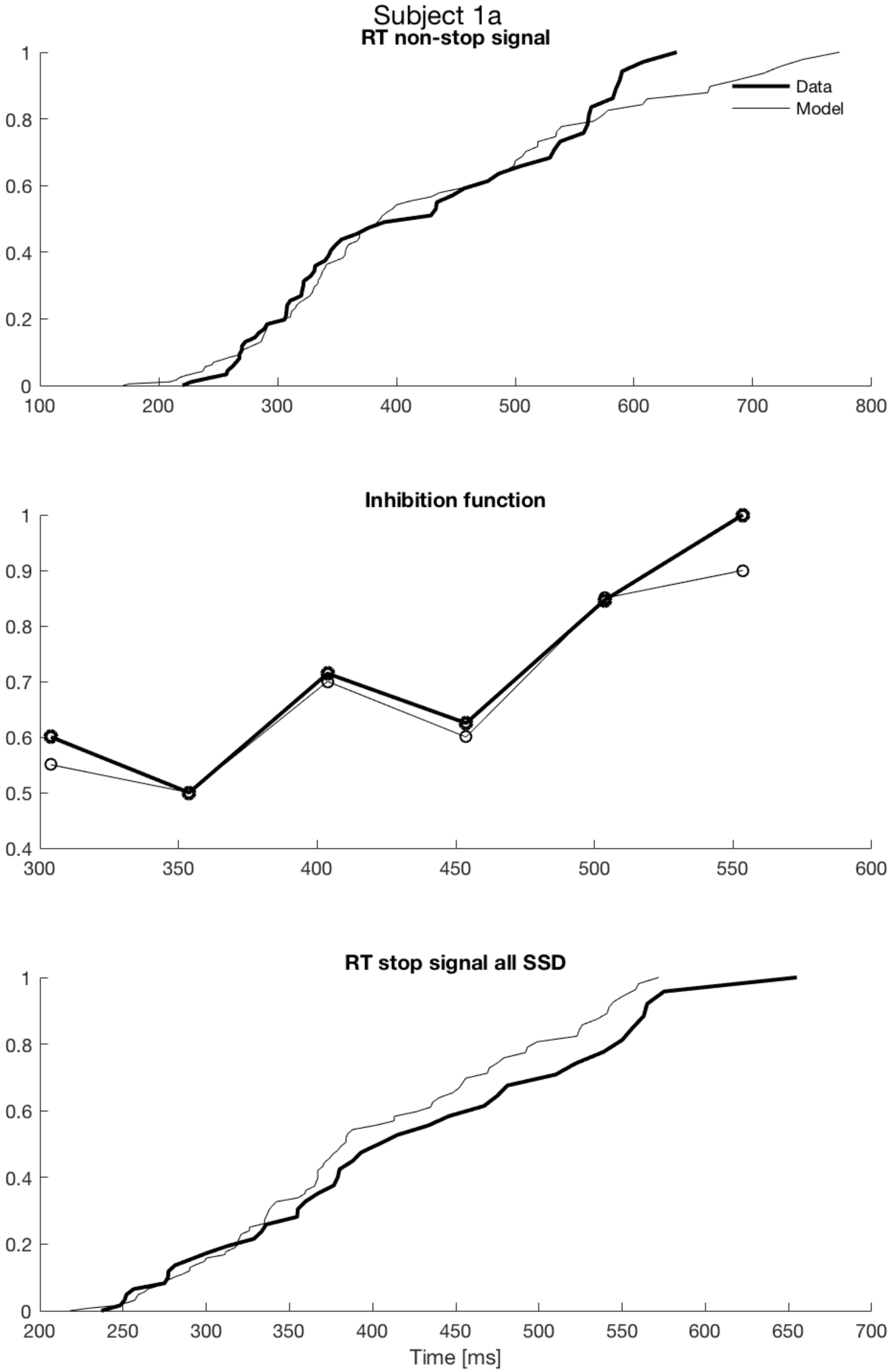

Subject 1a
RT non-stop signal
Data
Model
Inhibition function
RT stop signal all SSD
Time [ms]

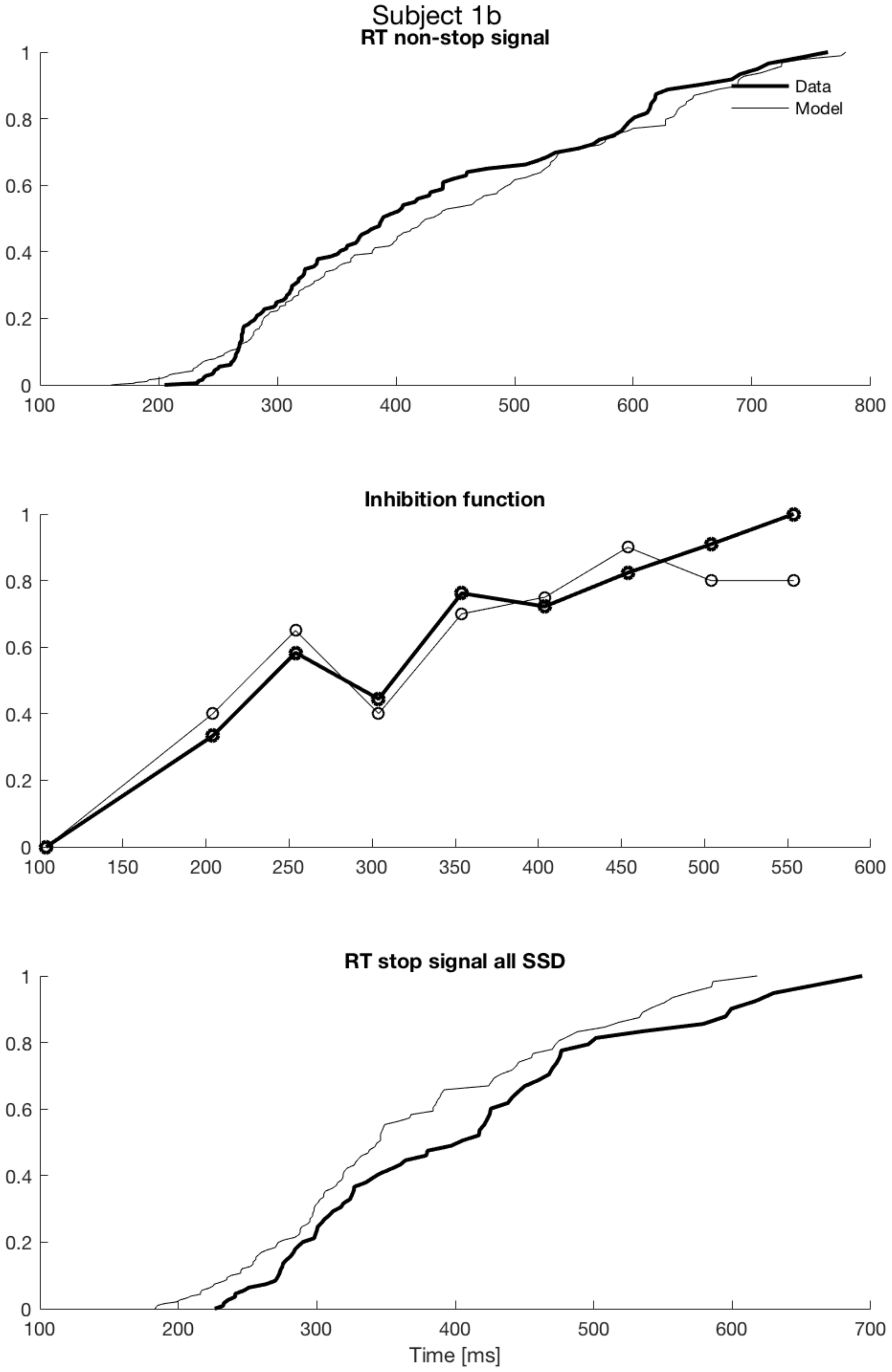

Subject 1b
RT non-stop signal
Data
Model
Inhibition function
RT stop signal all SSD
Time [ms]

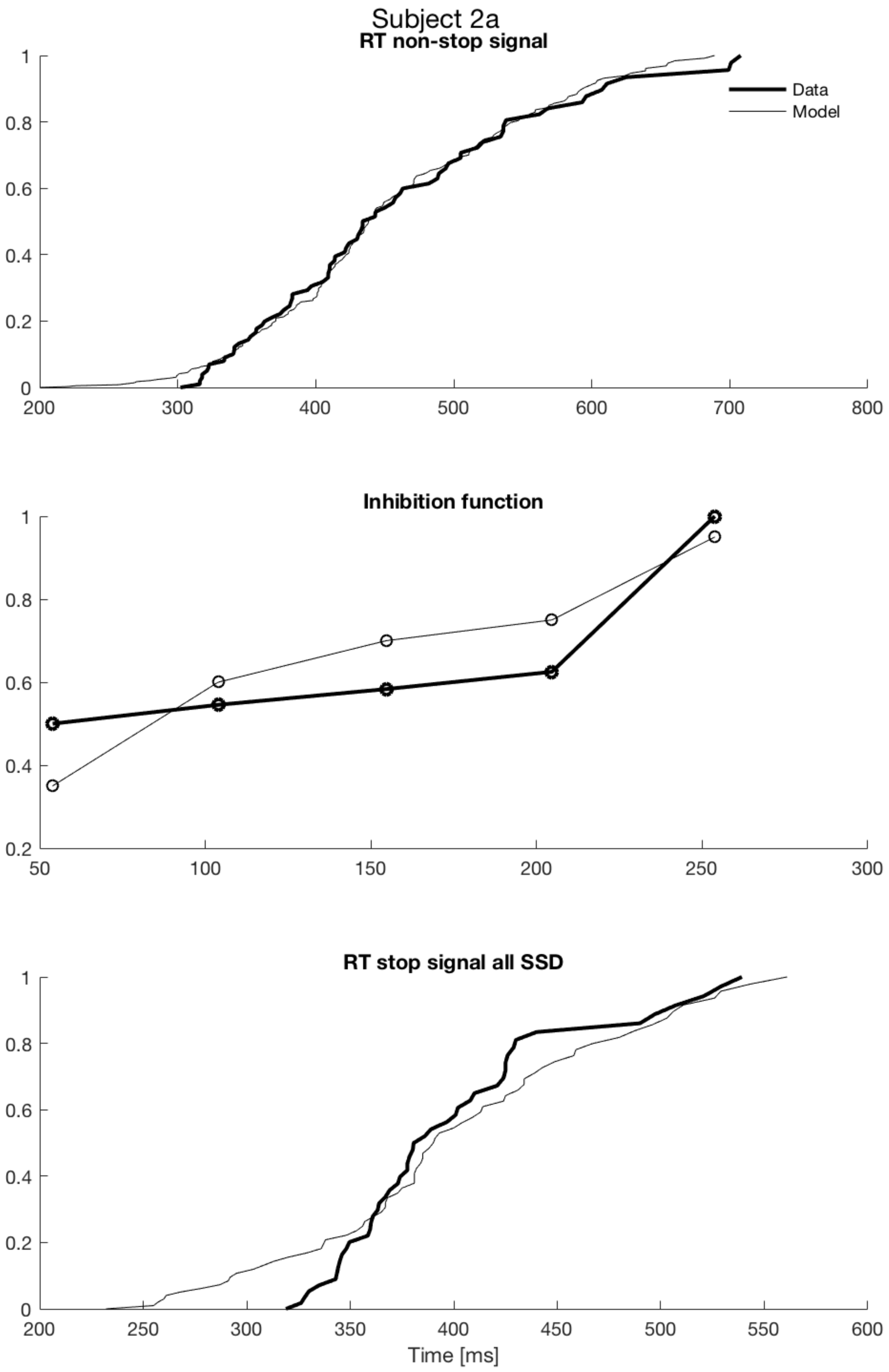

Subject 2a
RT non-stop signal
Data
Model
1
0.8
0.6
0.4
0.2
0
200
300
400
500
600
700
800
Inhibition function
1
0.8
0.6
0.4
0.2
50
100
150
200
250
300
RT stop signal all SSD
1
0.8
0.6
0.4
0.2
0
200
250
300
350
400
450
500
550
600
Time [ms]

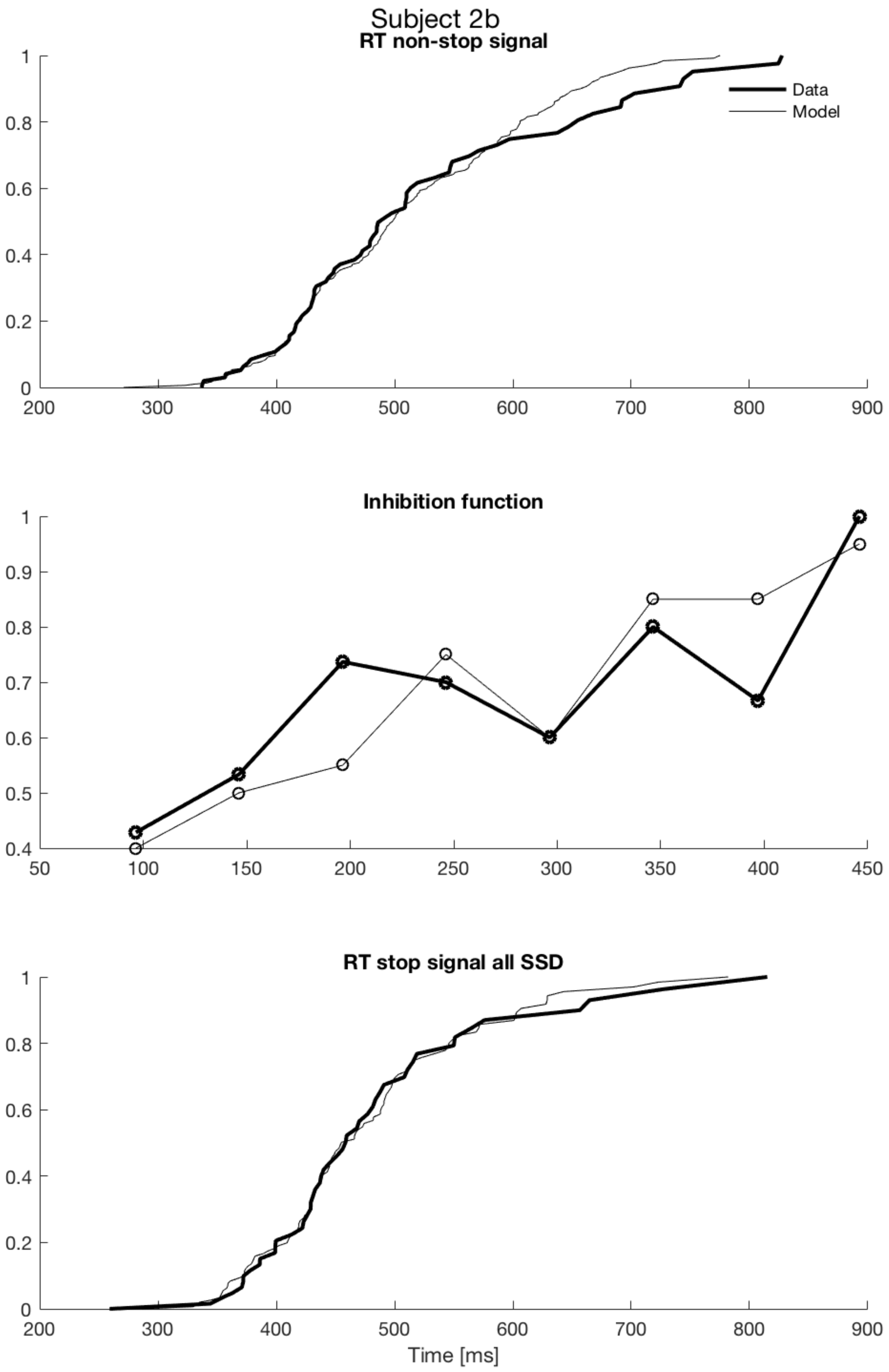

Subject 2b
RT non-stop signal
Data
Model
1
0.8
0.6
0.4
0.2
0
200
300
400
500
600
700
800
900
Inhibition function
1
0.9
0.8
0.7
0.6
0.5
0.4
50
100
150
200
250
300
350
400
450
RT stop signal all SSD
1
0.8
0.6
0.4
0.2
0
200
300
400
500
600
700
800
900
Time [ms]

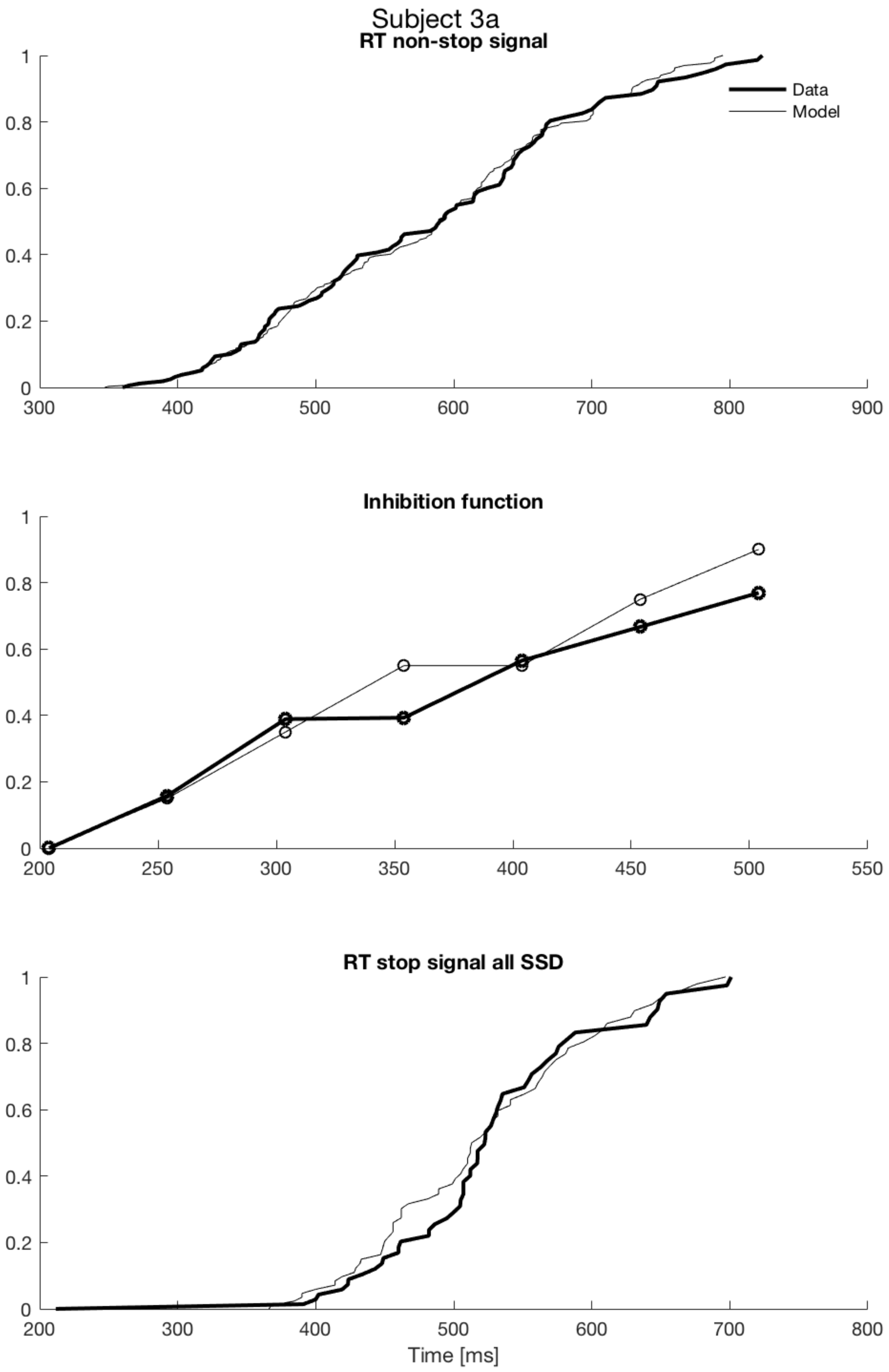
Subject 3a
RT non-stop signal
Data
Model
1
0.8
0.6
0.4
0.2
0
300
400
500
600
700
800
900
Inhibition function
1
0.8
0.6
0.4
0.2
0
200
250
300
350
400
450
500
550
RT stop signal all SSD
1
0.8
0.6
0.4
0.2
0
200
300
400
500
600
700
800
Time [ms]

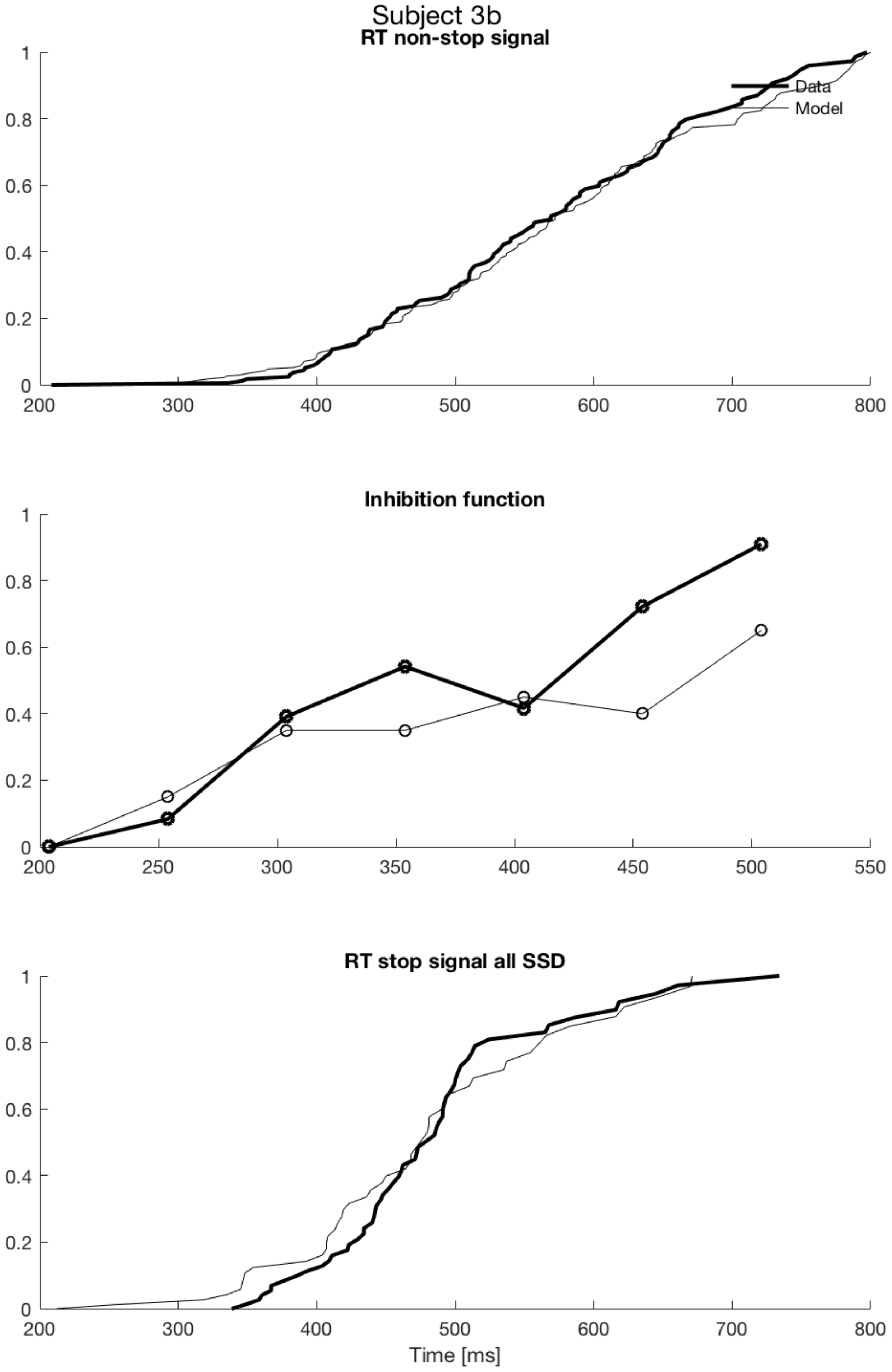

Subject 3b
RT non-stop signal
Data
Model
Inhibition function
RT stop signal all SSD
Time [ms]

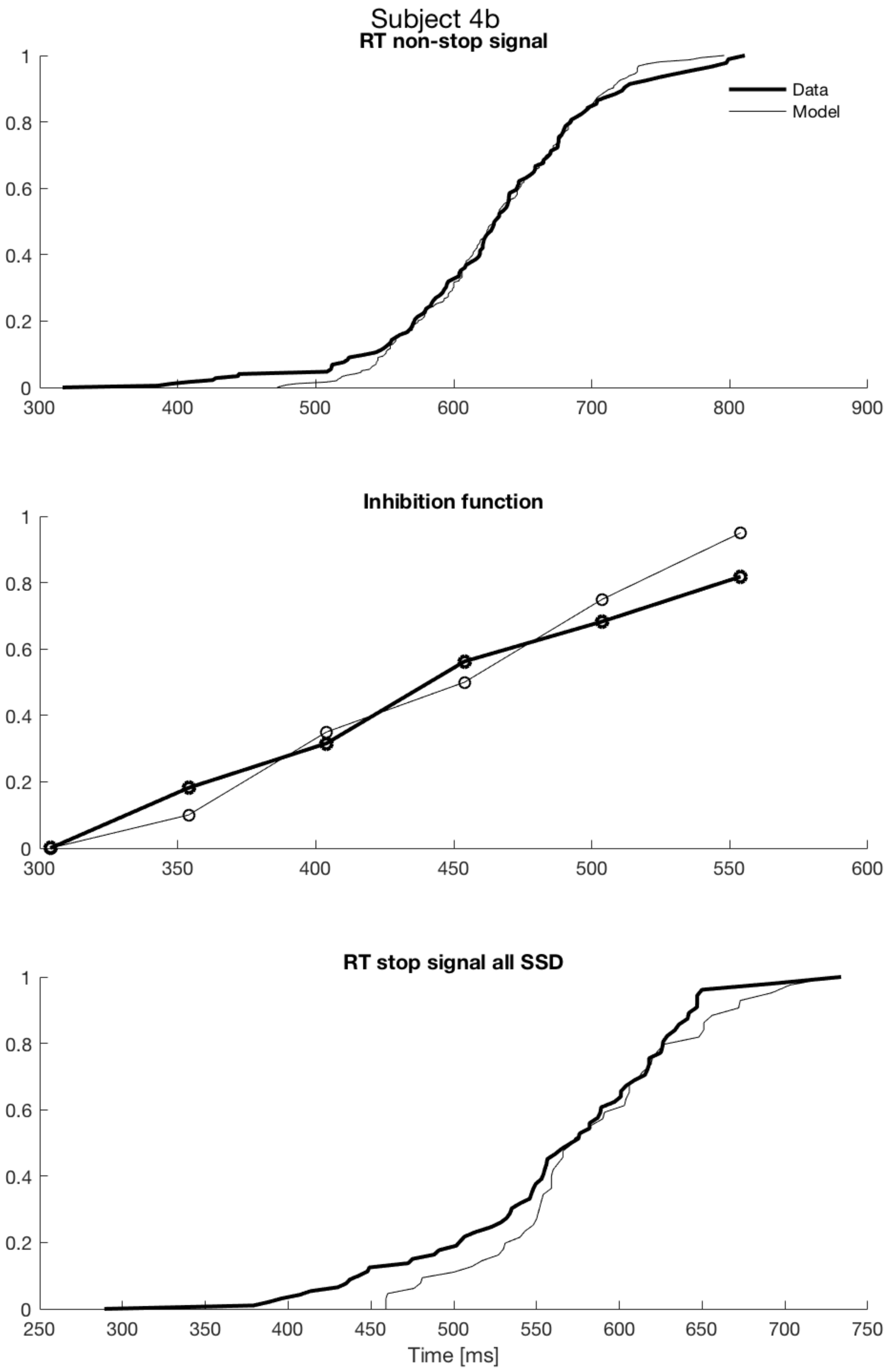

Subject 4b
RT non-stop signal
Data
Model
1
0.8
0.6
0.4
0.2
0
300
400
500
600
700
800
900
Inhibition function
1
0.8
0.6
0.4
0.2
0
300
350
400
450
500
550
600
RT stop signal all SSD
1
0.8
0.6
0.4
0.2
0
250
300
350
400
450
500
550
600
650
700
750
Time [ms]

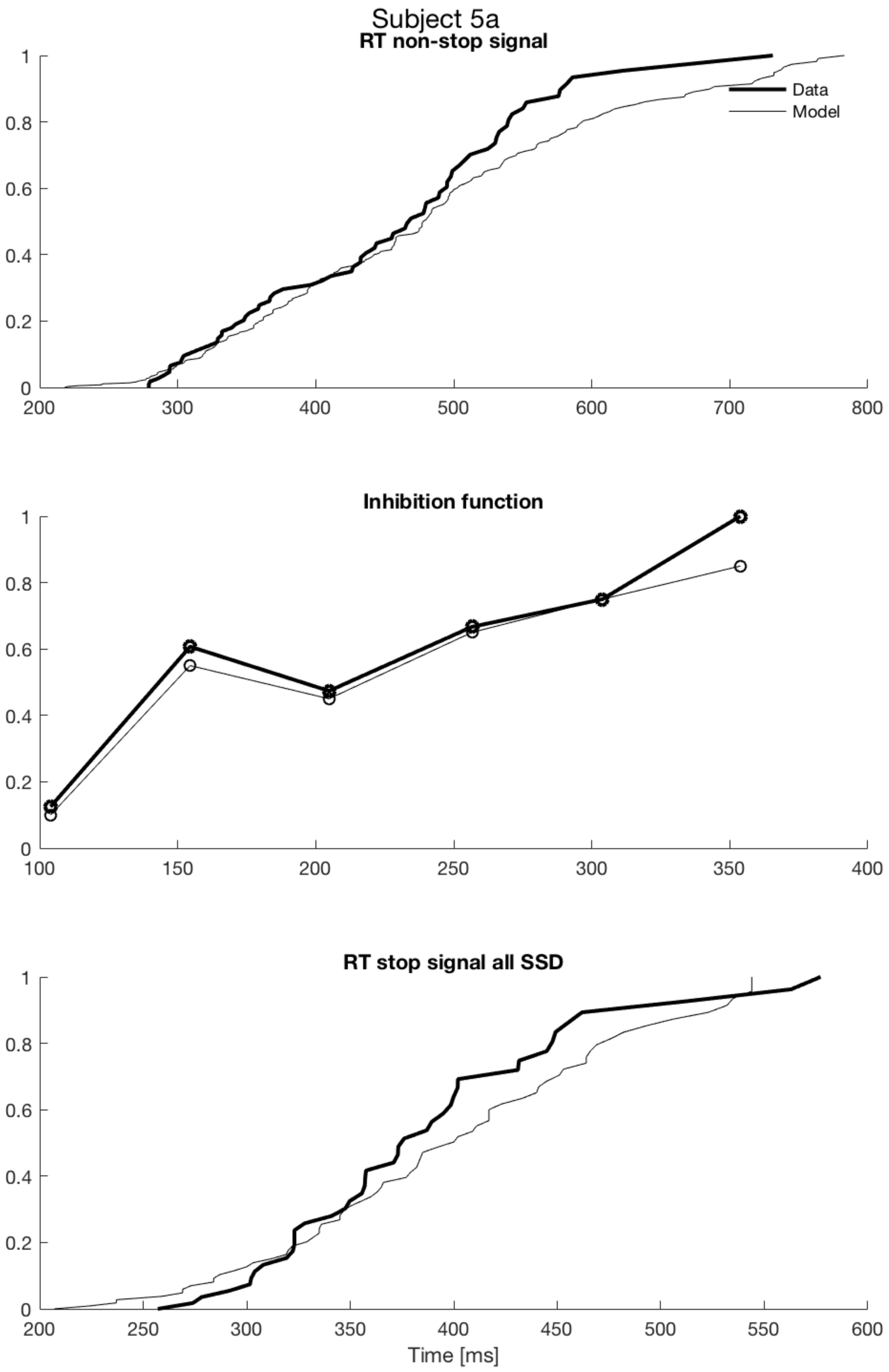

Subject 5a
RT non-stop signal
Data
Model
Inhibition function
RT stop signal all SSD
Time [ms]

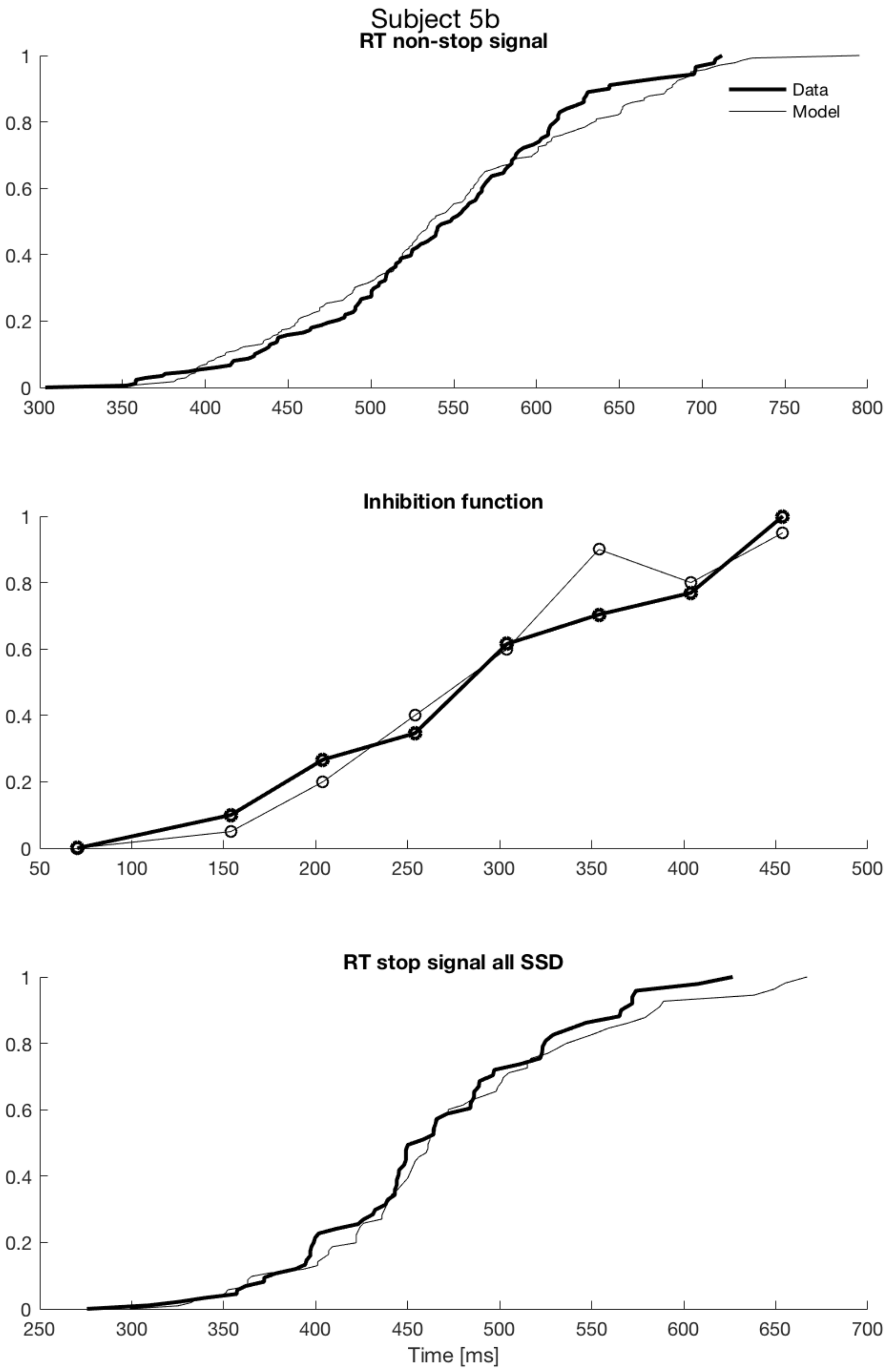
Subject 5b
RT non-stop signal
Data
Model
Inhibition function
RT stop signal all SSD
Time [ms]

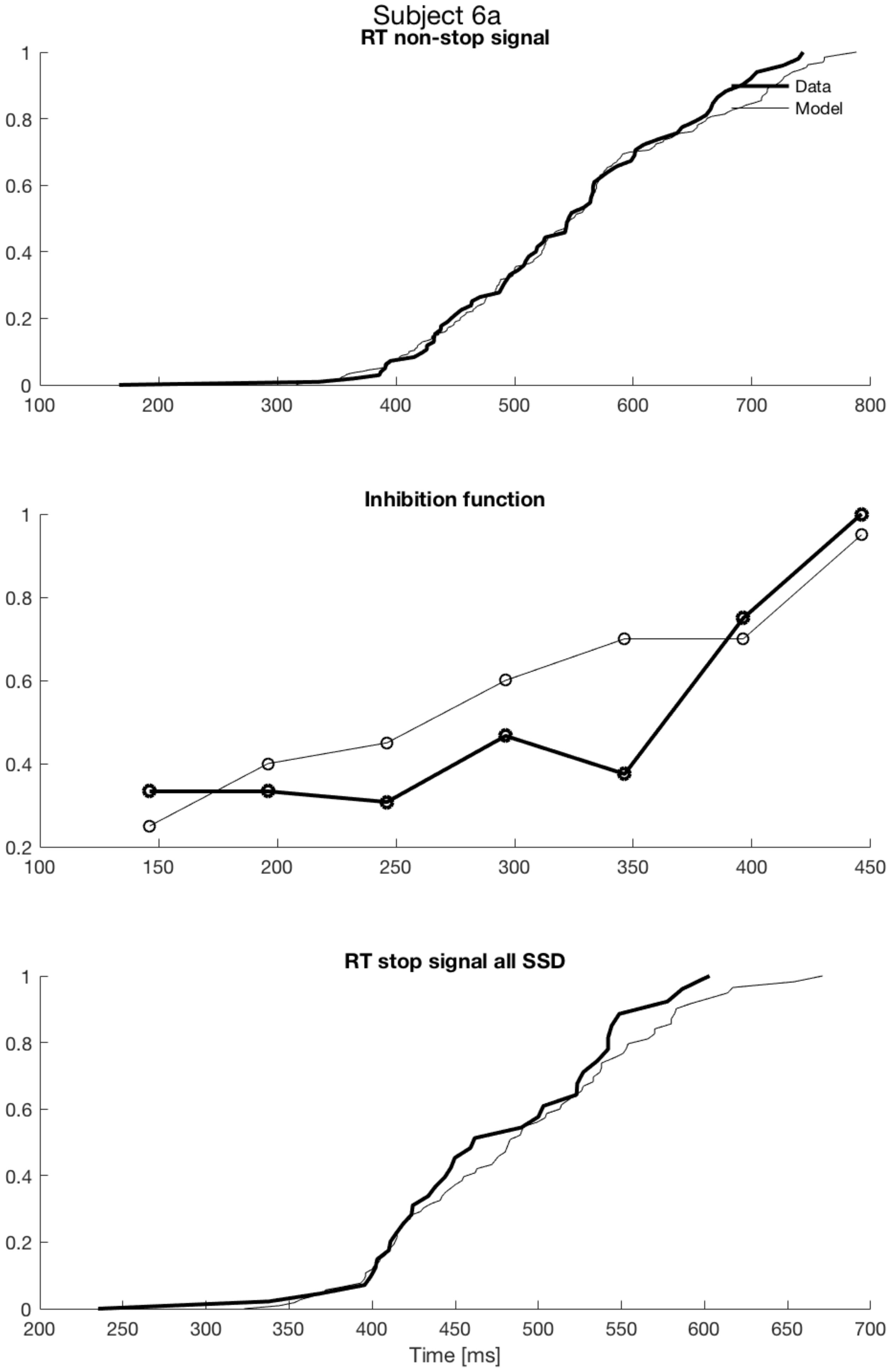

Subject 6a
RT non-stop signal
Data
Model
Inhibition function
RT stop signal all SSD
Time [ms]
